# Effect of autologous platelet-rich plasma on patients with acute type A aortic dissection underwent aortic arch replacement: A retrospective cohort study

**DOI:** 10.1371/journal.pone.0290384

**Published:** 2023-08-17

**Authors:** Xiaojin Wei, Kai Chen, Chaodong Huang, Kang Zhou, Ruixuan Wang, Yaping Wang, Yanying Xiao

**Affiliations:** 1 Department of Pain Management and Anesthesiology, The Second Xiangya Hospital, Central South University, Changsha, Hunan, China; 2 Department of Pain, Guizhou Provincial People’s Hospital, Guiyang, Guizhou, China; 3 Department of Cardiovascular Surgery, The Second Xiangya Hospital, Central South University, Changsha, Hunan, China; 4 Bourns Engineering, The University of California, Riverside, Riverside, CA, United States of America; AOU Policlinico ’Rodolico - San Marco’, ITALY

## Abstract

**Background:**

Coagulopathy and massive bleeding are common complications of patients with Stanford type A acute aortic dissection repair, and patients with these complications require many transfusions. Autologous platelet-rich plasma (PRP) is widely used to reduce the need for blood products. In the present study, we aimed to investigate the effects of PRP on blood conservation and the postoperative conditions of patients who underwent aortic arch replacement.

**Methods:**

Patients with aortic dissection undergoing aortic arch replacement were included initially application In all, 837 patients were divided into the PRP and non-PRP groups according to PRP use, whereupon a propensity score match was performed. The data analyzed included patient basic information, intraoperative information, postoperative biochemical examinations, and CTA reports.

**Results:**

In total, 610 patients were finally included (305 patients per group). Groups were well balanced after matching. Compared to the non-PRP group, less cryoprecipitate was transfused in the PRP group (10.0 [7.5, 11.0] vs. 10.0 [10.0, 11.5], *P* = 0.021), while no differences were found in packed RBC, FFP, and platelets between the two groups. Also, the surgery variables showed no differences. After surgery, patients in the PRP group showed higher postoperative serum albumin (36.43±4.20 vs. 35.39±4.40 g/L, *P* = 0.004) and total protein levels (59.38±6.25 vs. 58.06±7.19 g/L, *P* = 0.019) than the non-PRP group, but no significant differences in the levels of ALT, AST, Scr, and BUN. CTA reports showed that the proportion of patients with pleural effusion was lower in the PRP group (76.66% vs. 83.99%, OR = 1.59, 95% CI: 1.04–2.45, *P* = 0.028), while the proportions of pericardial effusion were not significantly different.

**Conclusions:**

PRP application in aortic arch replacement surgery reduced the transfusion of cryoprecipitate, increased the postoperative serum albumin and total protein levels, and reduced the incidence of pleural effusion. No effect of PRP application was found on other postoperative blood indicators and CTA reports.

## Introduction

Stanford type A acute aortic dissection (AAD) is a relatively rare but catastrophic vascular disease. It is defined as disruption of the medial layer provoked by intramural bleeding, resulting in separation of the aortic wall layers and subsequent formation of a true lumen and a false lumen with or without communication. Patients without treatment die at a rate of 1%–2% per hour on the first day, and within 48 hours almost 50% of patients present clinical symptoms, such as rupture, pericardial tamponade, valvular malfunction, and stroke [1, 2]. Treatment is challenging; conservative therapies showed markedly worse long-term outcomes than surgeries, while surgeries are complicated and result in high incidences of perioperative mortality (25%) and neurological complications (18%) [3]. Meanwhile, some anti-coagulation therapies might influence the severity and the outcomes of these patients [4–6].

Massive bleeding is a common and worrisome perioperative complication that requires compensative transfusion of blood products. Transfusion causes adverse effects on the body dependent on the amount transfused and increases the burden of health care resources. In this regard, various blood conservation methods have been explored in clinical practice, such as platelet-rich plasma (PRP) transfusion, blood salvaging systems, cell-saver techniques, and a minimized pump prime [7]. PRP is mainly applied in cardiovascular surgery for blood conservation. Harke was the first to attempt to re-transfuse platelets in extracorporeal circulation [8]. In the present retrospective clinical cohort study, we aimed to investigate (i) the effects of PRP on blood conservation and (ii) other effects on the postoperative conditions of patients who underwent aortic arch replacement.

## Materials and methods

### Study design, patient population and data collection

**Fig 1:**
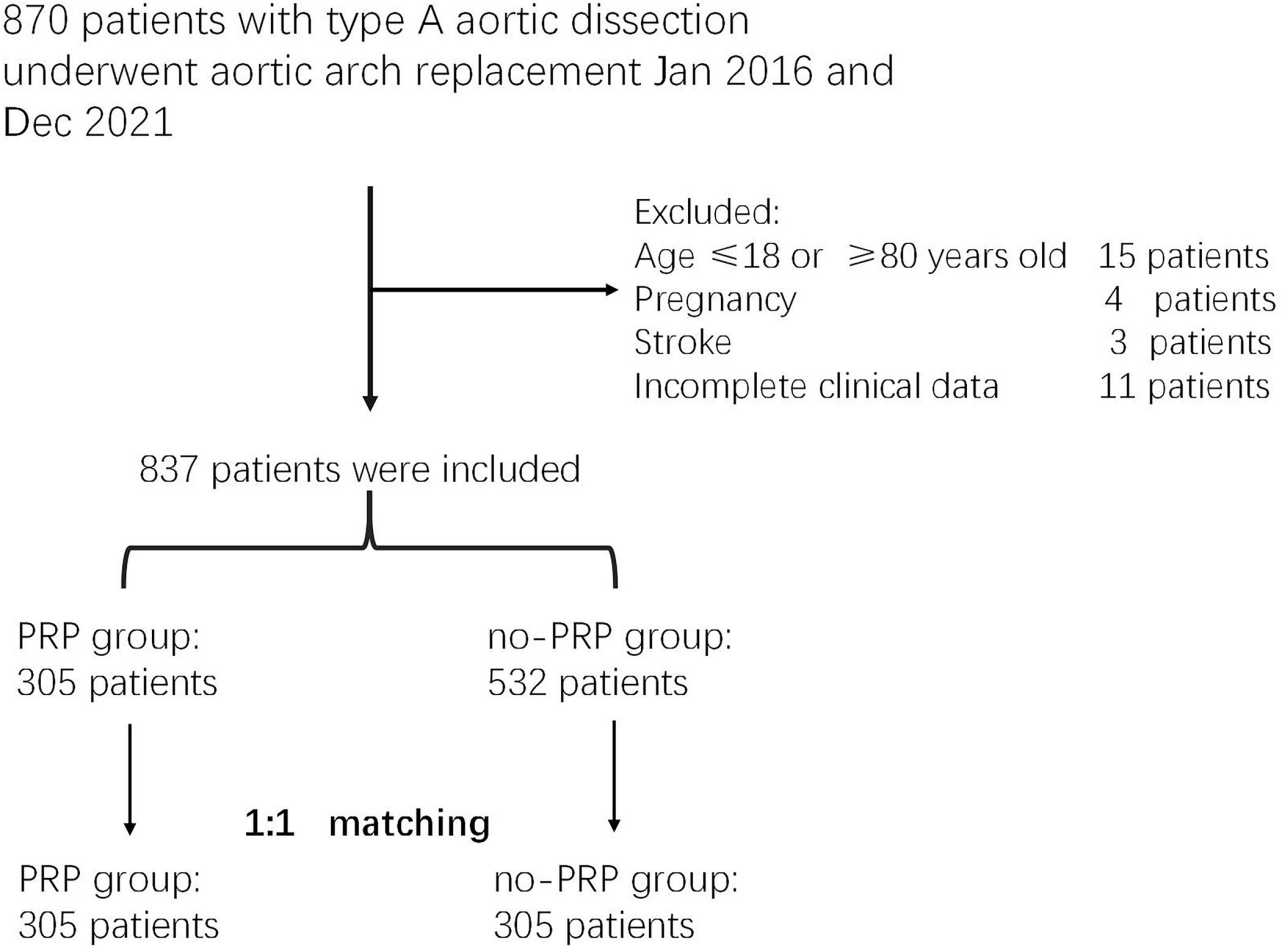
Study flow diagram.

This was a retrospective cohort study approved by the Internal Review Board of the Second Xiangya Hospital, Central South University, Changsha, Hunan Province, China (Ethical code: 2023EECRK001). The need for informed consent was waived because of the retrospective nature of the study. Patients with Stanford type A AAD were eligible if they were between 18 and 80 years of age and suitable for emergency surgery. A total of 870 consecutive patient records were collected between January 2016 and December 2021. Exclusion criteria were as follows: age below 18 or above 80 years, pregnancy, stroke, and incomplete data (Fig 1). Patients were divided into two groups based on PRP application during operation.

### Anesthesia routine

After routine anesthesia and intubation, general anesthesia was maintained with intravenous sufentanil, propofol, and neuromuscular blockade drugs. ECG, pulse oxygen saturation, left upper limb and lower limb arterial blood pressure, central venous pressure, nasopharyngeal and bladder temperature, and urine volume were monitored during the operation.

### PRP harvest technique

After anesthesia induction, whole blood (about 15–20 mL/kg) was collected via the right internal jugular vein using an autologous transfusion system (Cell saver elite harmonics corporation, USA). Ringer’s lactate and colloidal solution were injected into the peripheral vein for acute normovolemic hemodilution. The collected whole blood was divided into RBC and PRP by a machine. The whole process was completed before systemic heparinization. The isolated PRP was placed in a platelet oscillator with a frequency of 60 rpm at room temperature and transfused back into the patient after neutralizing heparin with protamin. Ringer’s lactate and colloidal solution were used to maintain the intravascular volume and hemodynamic stability during the PRP harvest, as well as continuous intravenous infusion of noradrenaline or dopamine. No cases of hemodynamic instability were noted during PRP collection. Isolated red blood cells can be transfused back to the patient at any time.

### Transfusion practice

The amount of allogeneic blood transfusion was determined by the surgeon, anesthesiologist, and perfusionist based on clinical parameters and TEG. Hemoglobin was maintained at approximately ≥7 g/dL during CPB and ≥8 g/dL after CPB and after the operation. Meanwhile, the amount of cryoprecipitate, FFP, and platelets was transfused during the operation according to the maximum amplitude, reaction time, and angle of the TEG.

### Statistical analysis

Patients with AAD underwent aortic arch replacement would receive different treatments according to their postoperative conditions. Therefore, we primarily analyzed the first results of the biochemical examination of blood and the CTA report to eliminate confounding factors of the consequence treatments.

Propensity score matching was performed to reduce the risk of confounding effects between the PRP and non-PRP groups. Patients were 1:1 matched according to covariates including sex, age, BMI, hypertension, smoking, alcohol consumption, and other factors (Table 1) using a logistic regression model with the k-nearest neighbor algorithm. After matching, an absolute standardized difference of <0.1 was considered as well balanced.

**Table 1:** Preoperative variables in the non-PRP and PRP groups.

Preoperative variable	non-PRP (*n* = 305)	PRP (*n* = 305)	Standardized mean differences	*P*-value
**Age**	49.95±11.96	49.78±10.53	1.57%	0.846
**Sex**	240 (78.69%)	237 (77.70%)	2.38%	0.769
**BMI**	26.37±4.53	26.06±4.45	6.92%	0.393
**Current smoker**	119 (39.02%)	115 (37.70%)	2.69%	0.740
**Alcohol consumption**	89 (29.18%)	98 (32.13%)	6.39%	0.430
**Diabetes**	9 (2.95%)	6 (1.97%)	6.34%	0.434
**Hypertension**	198 (64.92%)	194 (63.61%)	2.69%	0.740
**Previous cardiac surgery**	8 (2.62%)	8 (2.62%)	0	1
**COPD**	18 (5.90%)	21 (6.89%)	4.01%	0.620
**Liver diseases**	1 (0.33%)	1 (0.33%)	0	1
**Kidney diseases**	7 (2.30%)	9 (2.95%)	4.10%	0.613

Normally distributed data, including basic patient information, are presented as mean ± standard deviation and were analyzed using an unpaired *t*-test. Non-normally distributed data, including surgery duration, perioperative transfusions, and length of hospital stay, are presented as median with interquartile range and were analyzed with the Mann–Whitney *U* test. Categorical data, including the incidences of pleural effusion, are presented as numbers and percentages and were analyzed using the chi-squared test, and odds ratios (OR) with 95% confidence intervals (CIs) were calculated. Two-sided *P*-values of less than 0.05 were considered statistically significant. All analyses were performed with SPSS 24.0 (IBM, Armonk, NY, USA) and the Python statistical package (https://scipy.org).

## Results

### Baseline characteristics

**Fig 2:**
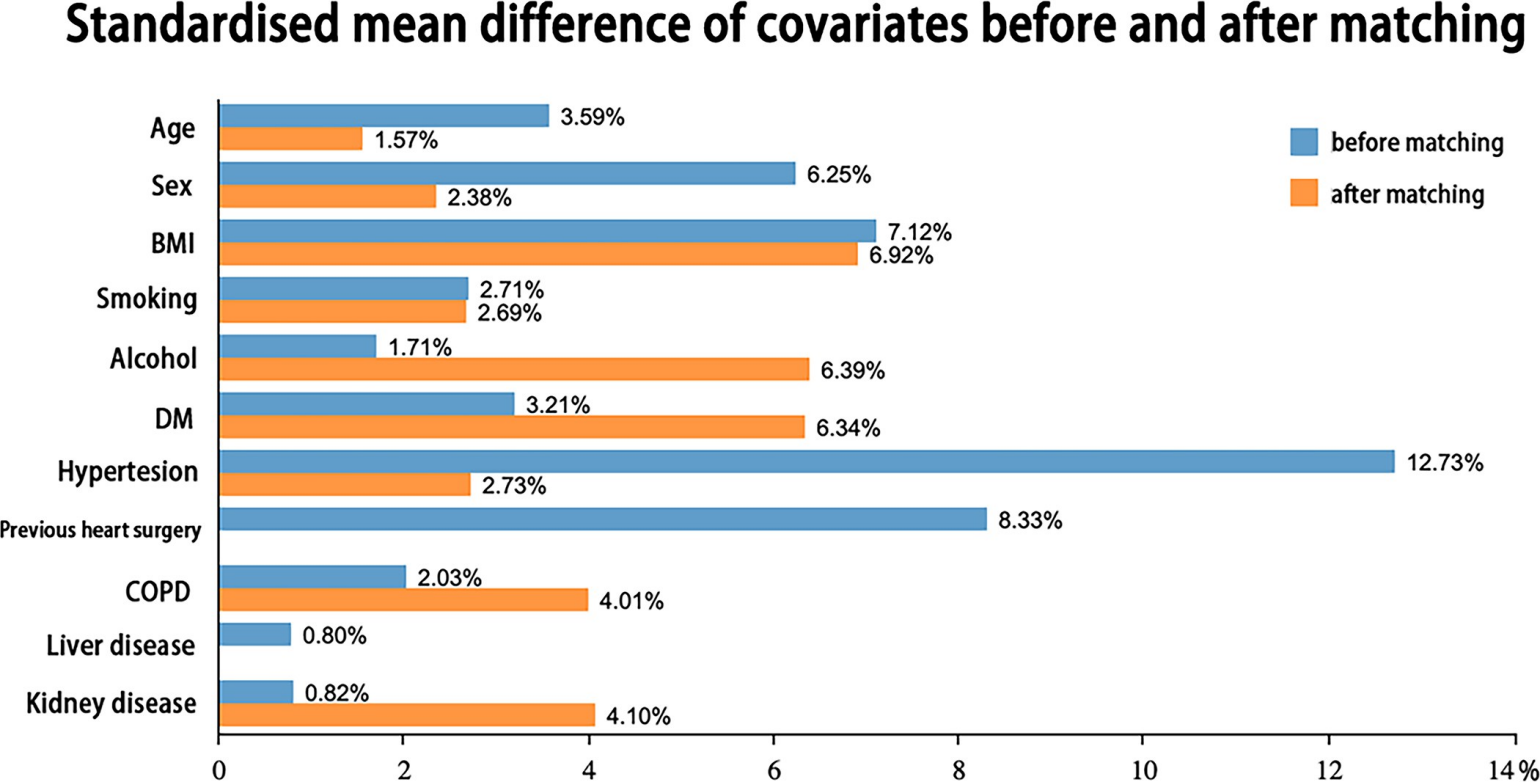
Standardized mean difference before and after matching.

After matching, a total of 610 patients with Stanford type A AAD were included. All standardized mean differences of covariates were less than 0.1 and simultaneously respective *P*-values were more than 0.05, indicating the patients in the two groups were well balanced (Fig 2, Table 1). The mean amount of PRP collected was 800 [665, 950] ml in the PRP group.

### Intraoperative data

As shown in Table 2, no significant differences in terms of duration of operation, CPB, aortic cross-clamping, and DHCA were observed between the two groups (*P* > 0.05). As for the transfusion of blood products, cryoprecipitate transfusion (u) was 10.0 [10.0, 11.5] in the PRP group, compared to 10.0 [7.5, 11.0] in the non-PRP group (*P* = 0.021). The transfusions of cell saver, packed RBC, FFP, platelets, and human albumin showed no significant differences between the two groups (*P* > 0.05). In arch surgery, the proportion of patients who underwent total arch or hemiarch replacement with or without CABG showed no statistical differences (*P* > 0.05) (Table 2).

**Table 2:** Perioperative variables in the non-PRP and PRP groups.

Variable	non-PRP (*n* = 305)	PRP (*n* = 305)	*P*-value
**Cryoprecipitate (u)**	10.0 (10.0, 11.5)	10.0 (7.5, 11.0)	0.021
**Cell saver (ml)**	1800 (1800, 2000)	1800 (1600, 2000)	0.493
**Packed RBC (u)**	4.0 (2.0, 6.0)	3.75 (2.0, 6.0)	0.348
**FFP (ml)**	350 (200, 400)	350 (200, 400)	0.993
**Platelets (u)**	1.0 (1.0, 1.0)	1.0 (1.0, 1.0)	0.171
**20% Human albumin (ml)**	100 (100, 100)	100 (100, 100)	0.348
**Surgery duration (min)**	503 (395, 592)	481 (383, 596)	0.998
**Bypass time (min)**	190 (117, 277)	206 (122, 297)	0.156
**Aortic cross-clamp time (min)**	96 (55, 147)	105 (72, 147)	0.276
**Circulatory arrest time (min)**	21 (16, 38)	23 (17, 37)	0.862
**Total arch replacement**	262 (85.90%)	274 (89.84%)	0.136
**Hemiarch replacement**	43 (14.10%)	31 (10.16%)	0.136
**CABG**	21 (6.89%)	26 (8.52%)	0.448

### Postoperative outcomes

Postoperative biochemical tests showed that the serum total protein level in the PRP group was significantly higher than in the non-PRP group (59.38±6.25 vs. 58.06±7.19, *P* = 0.019), and the serum albumin level was significantly higher in the PRP group than in the non-PRP group (36.43±4.20 vs. 35.39±4.40, *P* = 0.004). However, no significant differences in AST, ALT, TBIL, Scr, BUN, INR, APTT, and PT were found between the two groups (*P* > 0.05) (Table 3).

**Table 3:** Postoperative biochemical indicators in the non-PRP and PRP groups.

Postoperative clinical variable	non-PRP (*n* = 305)	PRP (*n* = 305)	*P*-value
**HB (g/L)**	99.79±14.11	99.79±13.72	0.996
**WBC (109/L)**	11.94±3.67	12.23±3.64	0.342
**PLT (109/L)**	144.18±74.88	136.82±71.70	0.223
**ALT (U/L)**	88.27±109.29	96.03±107.24	0.404
**AST (U/L)**	95.03±116.43	105.19±112.07	0.302
**TBIL (μM)**	27.47±25.15	29.72±39.96	0.422
**Total protein (g/L)**	58.06±7.19	59.38±6.25	0.019
**Albumin (g/L)**	35.39±4.40	36.43±4.20	0.004
**APTT (s)**	50.82±9.46	50.97±16.53	0.977
**PT (s)**	17.24±4.64	17.31±4.58	0.968
**INR**	1.29±0.57	1.33±0.42	0.771
**Scr (μM)**	114.06±78.92	126.71±110.26	0.114
**BUN (mM)**	10.57±2.84	11.05±2.94	0.075
**Hospital stay (days)**	19 (15.0, 24.0)	18.5 (15, 23)	0.860

Based on postoperative CTA reports, the proportion of patients with pleural effusion was 76.66% in the PRP group and 83.99% in the non-PRP group (OR = 1.59, 95% CI: 1.04–2.45, *P* = 0.028); the proportion of patients with pericardial effusion was not significantly different (71.43% in the PRP group vs. 70.11% in the non-PRP group, *P* = 0.729) (Table 4).

**Table 4:** Postoperative variables in the non-PRP and PRP groups from CTA reports.

Postoperative CTA	non-PRP (*n* = 281)	PRP (*n* = 287)	OR (95% CI)	*P*-value
**Pericardial effusion**	197 (70.11%)	205 (71.43%)	0.93 (0.65, 1.34)	0.729
**Pleural effusion**	236 (83.99%)	220 (76.66%)	1.59 (1.04, 2.45)	0.028

## Discussion

Platelet-rich plasma (PRP) is autologous serum containing not only high concentrations of platelets, but also abundant growth factors and cytokines, such as transforming growth factor-β (TGF-β), epidermal and vascular endothelial growth factors (EGF and VEGF), platelet-derived growth factor (PDGF), fibroblast growth factor (FGF), and insulin-like growth factor-1 (IGF-1). PRP is widely used, with applications ranging from orthopedic procedures and sports injuries to urology surgery and cardiac surgery [9].

Most recent studies demonstrated that PRP application in AAD surgery is an effective way for blood conservation in the perioperative period. In whole blood exposed to the CPB circuit, the platelets are activated, and coagulation factors are consumed during CPB. The use of PRP can maintain normal platelet function, preserve plasma volume, and ultimately reduce the transfusion volume [10]. But whether it can decrease postoperative complications and improve short-term outcomes is under debate. Some studies showed that PRP can decrease the length of ICU stay [11, 12], decrease the mean number of ventilator days [11–13] and the incidence of tracheostomy [11, 13], and decrease the length of hospital stay [12, 14], while another study showed that PRP application might increase the risk of postoperative acute kidney injury, without decreasing the length of hospital stay or in-hospital mortality [10].

Consistent with the abovementioned studies, we found that the incidence of perioperative cryoprecipitate transfusion was reduced in the PRP group compared to the non-PRP group. Unexpectedly, we observed no significant differences in other blood product transfusions between the two groups, including packed red blood cells and fresh frozen plasma. The differences from previous studies may result from different blood transfusion standards and programs in the different centers. Additionally, we made the following observations.

### 1) Increased serum albumin and total protein levels in the first postoperative examination

Serum albumin levels are decreased after cardiac surgery due to surgical injury, blood loss, systematic inflammation, hypermetabolism, and so on [15, 16]. Serum albumin levels serve as a marker of the host response to a severe operative insult [17]. In patients with low serum albumin levels, complication and mortality rates after CABG or other cardiac surgeries are high [15, 18, 19]. As for patients with AAD, a recent study showed that albumin levels are associated with a high risk of in-hospital mortality [16].

In PRP which is re-transfused to the body, the albumin concentration is nearly the same as in blood and therefore PRP application can increase serum albumin. Additionally, high concentrations of platelets and growth factor in PRP (TGF-β, VEGF) may also play a key role by stimulating epithelial and endothelial cell regeneration, boost angiogenesis and collagen deposition, and consequently accelerate the healing process [20, 21]. Better wound healing of surgical incisions and tears can reduce serum albumin leakage or exudation to the extravascular space. At the same time, various growth factors in PRP were proven to exert some modulatory effects on acute and chronic inflammation [20]. For example, TGF-β was reported to prevent excessive leukocyte recruitment at the lesion sites [22]. and HGF seems to have a crucial anti-inflammatory function by inhibiting NF-κB signaling [23]. By regulating and inhibiting excessive inflammation, PRP can reduce systematic protein consumption and thus slow down albumin catabolism in the perioperative period. Additionally, some other growth factors (such as IGF-1) in PRP were reported to promote protein synthesis and increase serum albumin and total protein levels in some liver diseases or injuries [24–26]. Interestingly, we found that serum total protein showed the same trends as serum albumin. Taken together, albumin in PRP, reduced protein degradation, reduced protein catabolism, and increased protein production may also contribute to the increase in serum albumin levels.

### 2) The proportion of patients with pleural effusion is reduced

Pleural effusion demonstrated by serial CT is a common finding in patients with AAD (87.5%) [27]. It is a common complication of cardiac surgery, and it is associated with postoperative mortality and significant resource consumption [28]. The most common conditions that result in effusion are cardiac failure, pneumonia, and increased pleural membrane permeability [29].

In the present study, the proportion of patients with pleural effusion in the first postoperative CTA report was significantly lower in the PRP group than in the non-PRP group. This may be explained as follows. Better surgical wound healing and higher plasma colloid oncotic pressure due to increased serum albumin levels can reduce fluid leakage or exudation into the pleural space. Furthermore, recent studies demonstrated that PRP attenuated these cardiac pathological changes by exerting anti-inflammatory effects and promoting cardiomyocyte repair in high-dose isoproterenol-induced cardiotoxicity and LPS-induced cardiac injury rat models [30, 31]. Mishra found that PRP-treated mice had a greater left ventricular ejection fraction after undergoing ischemia and ischemia–reperfusion than PBS controls [32]. Furthermore, after acute myocardial infarction for 8 weeks, PRP was reported to structurally and functionally improve the injured heart muscle in pigs [33]. Therefore, PRP may mediate the repair and regeneration of cells in the early stage of cardiac injury and thus improve cardiac output, which may play a key role in decreasing the incidence of postoperative pleural effusion.

However, there were no differences in the proportion of patients with pericardial effusion between the two groups, though the association between pericardial and pleural effusion has been previously reported in studies primarily investigating pericardial effusion [28].

### 3) There were no differences in other postoperative outcomes between the PRP and non-PRP groups

Although the mean levels of Scr and BUN were higher in the PRP group than in the non-PRP group, no statistically significant differences were found. These results contrast with those of Tong, who showed that patients in the PRP group showed higher Scr and lactic acid levels, which were associated with a higher incidence of acute kidney injury on postoperative day 1–3 [10]. The diversion may come from different transfusion amounts, anesthesia routines, or operation details. Simultaneously, the results showed no differences in AST and ALT levels, which serve as markers of acute liver injury, between the two groups.

## Conclusion

Because of high platelet concentrations and various growth factors, PRP application in aortic arch replacement surgery reduced the transfusion of cryoprecipitate, increased serum albumin and total protein levels, and reduced the incidence of pleural effusion. No effects of PRP application on other postoperative blood biochemical indicators and CTA reports were found.

## Limitations

Surgery in the two groups was performed by different surgical teams, and the duration of operation, bypass time, aortic cross-clamp time, and circulatory arrest time were different. Although the surgical operations and perioperative management of the two groups were consistent, the results may still be affected. Intraoperative transfusion of allogeneic blood products is a clinical decision with a degree of subjectivity, although perioperative transfusion indications are specified. This study is a single-center retrospective study and requires a multi-center prospective study for validation.
